# Enzymatic and Pro-Inflammatory Activities of *Bothrops lanceolatus* Venom: Relevance for Envenomation

**DOI:** 10.3390/toxins9080244

**Published:** 2017-08-07

**Authors:** Marie Delafontaine, Isadora Maria Villas-Boas, Laurence Mathieu, Patrice Josset, Joël Blomet, Denise V. Tambourgi

**Affiliations:** 1 Prevor Laboratory, Moulin de Verville, Valmondois 95760, France; mdelafontaine@prevor.com (M.D.); lmathieu@prevor.com (L.M.); jblomet@prevor.com (J.B.); 2 Immunochemistry Laboratory, Butantan Institute, São Paulo 05503-900, Brazil; isadora.boas@butantan.gov.br; 3 Trousseau Hospital, Paris 75012, France; patricejosset@gmail.com

**Keywords:** snake venom, *Bothrops lanceolatus*, toxic activities, antivenom

## Abstract

*Bothrops lanceolatus*, commonly named ‘Fer-de-Lance’, is an endemic snake of the French Caribbean Island of Martinique. Envenomations by *B. lanceolatus* present clinical aspects characterized by systemic thrombotic syndrome and important local inflammation, involving edema and pain but limited hemorrhage. To investigate mechanisms of venom-induced inflammation, *B. lanceolatus* venom was characterized, its cross-reactivity with bothropic antivenom explored, its cytotoxicity on human keratinocytes and vascular cells, and the production of cytokines and chemokines were analyzed. We used electrophoretic separation, zymography, colorimetric or fluorimetric enzymatic assays, and immunochemical assays. Therapeutic South American bothropic antivenom cross-reacted with *B. lanceolatus* venom and completely or partially abolished its PLA2, hyaluronidase, and proteolytic activities, as well as its cytotoxicity for keratinocytes. The substrate specificity of *B. lanceolatus* venom proteases was emphasized. *B. lanceolatus* venom cytotoxicity was compared to the *B. jararaca* venom. Both venoms were highly cytotoxic for keratinocytes (HaCaT), whereas *B. lanceolatus* venom showed particularly low toxicity for endothelial cells (EAhy926). Patterns of cytokine and chemokine production by cells exposed to the venoms were highly pro-inflammatory. Thus, the results presented here show that *B. lanceolatus* venom toxins share important antigenic similarities with South American *Bothrops* species toxins, although their proteases have acquired particular substrate specificity. Moreover, the venom displays important cytotoxic and pro-inflammatory action on human cell types such as keratinocytes and endothelial cells, which are important players in the local and systemic compartments affected by the envenomation.

## 1. Introduction

In South and Central America, *Bothrops* species account for the majority of venomous ophidian accidents. Envenomation by these snakes has a complex pathophysiology, characterized by prominent local effects (edema, pain, hemorrhage, and necrosis) and systemic effects such as coagulation disturbances, hemorrhage, and renal failure [1,2]. *Bothrops lanceolatus*, commonly known as ‘Fer-de-lance’, is an endemic species confined to the island of Martinique in the Caribbean, where it is responsible for an average of 30 cases of human envenomati*on per* year [3]. *B. lanceolatus* venom induces local and systemic effects comparable to bothropic syndrome, but the envenomation is characterized by a predominant prothrombotic profile. Systemic thrombosis development can lead to cerebral, myocardial, or pulmonary infarctions that only rapid treatment with the monospecific commercial antivenom (Bothrofav^®^, Sanofi-Pasteur, France) can prevent [2,3,4,5].

*B. lanceolatus* venom contains five major types of enzymes; zinc-dependent snake venom metalloproteases (SVMPs), snake venom serine proteinases (SVSPs), phospholipases A2 (PLA2), L-amino acid oxidases, and a specific C-type lectin-like molecule [6,7]. SVMPs are particularly abundant in *Bothrops* venoms [8,9]. They all share a highly conserved zinc-dependent active site but vary in their non-catalytic domains composition, an important factor in the classification of these toxins [10]. Class I SVMPs (PI-SVMPs) are only composed of the catalytic protease domain. Class II proteins also present a disintegrin domain, whereas class III SVMPs have both a disintegrin-like and a cysteine-rich domain. In addition to the class III structure, some PIII-SVMPs present two C-type lectin-like domains [11,12]. SVMPs induce hemorrhage, myonecrosis, cutaneous lesions and inflammation, and degradation of coagulation factors and extracellular matrix components. Through their non-catalityc domains, they interfere with platelet functions and cell adhesion molecules [13]. SVSPs have a serine residue in their active site. They were shown to disturb haemostasis by affecting platelet function and degrading coagulation cascade components [14]. PLA2s hydrolyse glycerophospholipids in the sn-2 position. They are present in enzymatically active or inactive forms in *Bothrops* venoms. They display a wide range of biological effects such as neurotoxicity, cardiotoxicity, myotoxicity, hemolysis, and, anti-coagulating, anti-platelet, and edema-forming activities [15]. Three enzymes have already been purified and characterized from *B. lanceolatus* venom; a hemorrhagic PI-SVMP, an acidic phospholipase, and a potent fibrinogenolytic enzyme [16,17].

Inflammation induced by *B. lanceolatus* venom has been described in rat and mouse models [18,19,20,21]. *B. lanceolatus* venom-induced edema is accompanied by local hemorrhage, involves neutrophil infiltration, mast cell degranulation, and the release of arachidonic acid metabolites, bradykinin, histamine, and serotonin in the first hours after inoculation [18,19,21]. Intraperitoneal injection of the venom was followed by intense neutrophil migration, a chemotactic process in which macrophages and lipoxygenase metabolites were involved [20]. Intravenous administration of the specific commercial antivenom shows low efficacy at inhibiting venom-induced rat hind paw edema [19].

The mechanism of thrombosis observed in envenomation by *B. lanceolatus* has not yet been elucidated. The venom cleaves purified human fibrinogen but is unable to clot citrated human plasma, and almost normal coagulation profile can be observed in patients developing thrombosis, suggesting that the thrombosis mechanism could involve vascular endothelial cells or platelet activation [2]. It is deprived of defibrinating activity in mice [22,23]. The venom thrombotic syndrome observed in cases of human envenomations is not reproducible in mice [6].

Like the monospecific antivenom raised against *B. lanceolatus* venom, the therapeutic Costa Rican polyvalent antivenom (Clodomiro Picado Institute, Costa Rica) was shown to recognize venom toxic compounds in vitro and to be fully effective in mice in neutralizing the lethal, hemorrhagic, edema-forming, myotoxic, and indirect hemolytic activities of the venom [6,22]. The commercial Brazilian bothropic antivenom is obtained from the hyperimmune sera of horses immunized with a pool of five Brazilian bothropic venoms: *Bothrops jararaca* (50%), *Bothrops alternatus* (12.5%), *Bothrops jararacussu* (12.5%), *Bothrops moojeni* (12.5%), and *Bothrops neuwiedi* (12.5%). This antivenom cross reacts with *B. lanceolatus* venom and its hemorrhagic PI-SVMP, BlaH1, and neutralizes the hemorrhagic activity of this toxin [17].

The main *B. lanceolatus* venom activities have been studied [7,15,16,17,18,22,24]. Here, we extended the characterization of *B. lanceolatus* venom enzymatic and cytotoxic activities to improve the understanding of the venom-induced local inflammation mechanism. The venom enzymatic activities were confirmed and further investigated. The antivenom cross-reaction and the inhibition of in vitro *B. lanceolatus* venom enzymatic activities were studied to highlight antigenic similarities between *B. lanceolatus* and South American *Bothrops* species venoms. To approach human inflammation mechanisms, we studied *B. lanceolatus* venom cytotoxicity on human cell lines of keratinocytes and vascular endothelial cells and cytokines/chemokines production upon venom-exposure.

## 2. Results

### 2.1. Immunochemical Characterization and Cross-Reactivity of B. lanceolatus Venom

**Figure 1:**
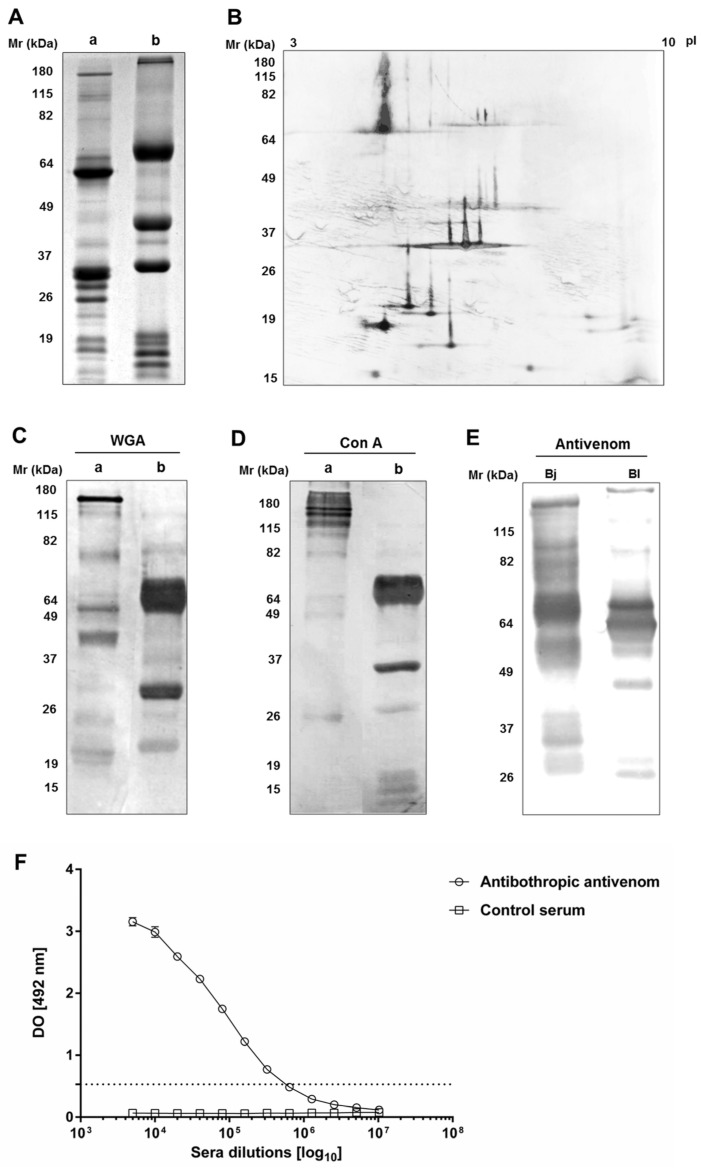
Uni- and bi-dimensional electrophoretic analysis of *B. lanceolatus* venom, its cross-reaction with bothropic antivenom, and the presence of glycosylated proteins in the venom. (**A**,**C**,**D**) Samples of *B. lanceolatus* venom were submitted to electrophoretic SDS-PAGE separation (12% of acrylamide) in non-reducing (**a**) and reducing (**b**) conditions. (**A**) The gel on which 30 µg of venom was separated was stained with Coomassie Blue R-250. (**B**) Strips with pH gradient (3 to 10) were rehydrated with buffer containing *B. lanceolatus* venom (100 µg) before isofocalization. After washing with reducing and chelating buffers, the focalized strips were submitted to SDS-PAGE electrophoresis (12% of acrylamide). The resulting gels were silver stained. (**C**,**D**) Venom samples (15 µg) were separated by electrophoresis and electrotransferred to nitrocellulose membranes. The membranes were incubated with the peroxidase-conjugated lectins, WGA and Con A. Recognized bands were visualized with DAB. (**E**) After electrophoresis in non-reducing conditions, samples (5 µg) of *B. jararaca* (Bj) and *B. lanceolatus* (Bl) venoms were electrotransferred to nitrocellulose membranes and incubated with bothropic antivenom diluted 1:10,000 followed by GAH/IgG-AP (1:7500). Cross-reacting bands were visualized with NBT-BCIP. (**F**) ELISA plates were coated with *B. lanceolatus* venom (1 µg/well). They were incubated with serial dilutions of bothropic antivenom or botulinum toxin antiserum, as negative control, followed by GAH/IgG-AP (1:3000). The results showed are representative of two experiments, realized in duplicates. The titer was determined as the highest antivenom dilution, which produced an absorbance eight times greater than the absorbance determined for the control serum. This absorbance value is represented by a dotted line.

The electrophoretic profile analysis of *B. lanceolatus* venom in non-reducing conditions reveals the presence of proteins with molecular masses between 10 and 180 kDa (Figure 1(Aa)). In reducing conditions, the venom’s profile consists of three major bands, the molecular masses of which are 70 and 37 kDa (Figure 1(Ab)). The bidimensional electrophoresis of the venom (Figure 1B) revealed that the majority of its components are acidic, with an isoelectric point (pI) between 4 and 7 and with only one isoform. Some basic proteins were revealed with *M_r_* between 15 and 19 kDa. A protein with a molecular mass of 30 kDa presented three isoforms, with an pI between 6.6 and 6.9.

The lectin western blot analysis shows that several proteins contain, *N*-acetyl-d-glucosamine and/or sialic acid and terminal α-d-mannosyl and/or α-d-glucosyl groups, as determined using WGA and Con A, respectively (Figure 1C,D).

The cross-reactivity of the venom with bothropic antivenom was validated by two methods; western blotting and ELISA, using *B. jararaca* venom as the positive control, with botulinum toxin antiserum as the negative control. Figure 1E shows a strong recognition of several components of *B. lanceolatus* venom by the bothropic antivenom, mainly proteins with *M_r_* between 26 and 70 kDa. By ELISA, the ability of the bothropic antivenom to recognize *B. lanceolatus* venom was confirmed with a 1:320,000 antibody titer (Figure 1F). The control antiserum did not recognize any of the venom components, neither by western blot (data not shown) nor by ELISA (Figure 1F).

### 2.2. Enzymatic Activities of B. lanceolatus Venom

The hyaluronidase activity of the venom was determined using hyaluronic acid as the substrate and *B. jararaca* venom as the positive control. The specific hyaluronidase activity of *B. lanceolatus* venom was 20.5 ± 2.4 TRU/mg, which was about five times lower than the *B. jararaca* venom activity in the same conditions (Table 1). A fluorimetric assay was used to study the presence of PLA2 activity in the venom, using *B. jararaca* and *C. durissus terrificus* venoms as positive controls. The specific PLA2 activity of *B. lanceolatus* venom reached 231.3 ± 6.4 UF/min/µg, which was significantly higher than that of *B. jararaca* venom but lower than *Crotalus* venom (Table 1).

**Figure 2:**
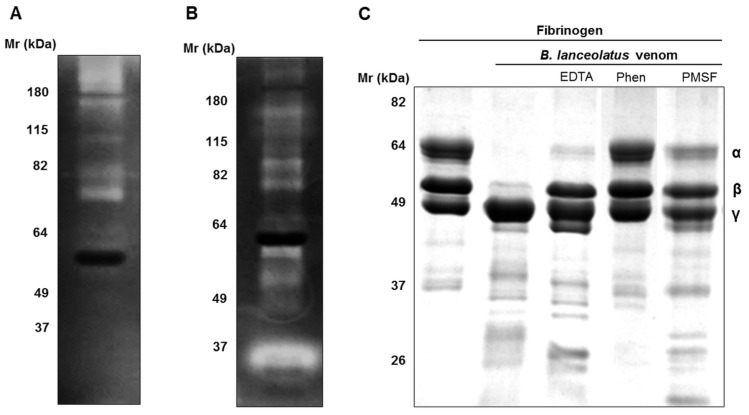
Gelatinolytic and fibrinogenolytic activities of *B. lanceolatus* venom. (**A**,**B**) Samples of *B. lanceolatus* venom (25 µg) were separated by SDS-PAGE in 12% acrylamide gels containing (**A**) 10% gelatin or (**B**) 10% fibrinogen under non-reducing conditions at 4 °C. The gels were then incubated overnight at 37 °C in substrate buffer (pH 8.3) and stained with Coomassie Blue R250. (**C**) Samples of fibrinogen (30 µg) were incubated with *B. lanceolatus* venom (0.5 µg) for 1 h at 37 °C in the absence or presence of proteases inhibitors (20 mM), EDTA, 1,10-phenanthroline (Phen), or PMSF. Samples were then separated by SDS-PAGE electrophoresis in reducing conditions before Coomassie Blue R250 staining.

*B. lanceolatus* venom (25 µg) was submitted to gelatin and fibrinogen zymography. Figure 2A exhibits the presence of high molecular mass gelatinases (*M_r_* > 115 kDa), as well as smaller ones, between 64 and 85 kDa. Several proteins are able to cleave fibrinogen with molecular masses between 20 and 180 kDa (Figure 2B). However, a band of about 30 kDa presents higher activity, compared with the other proteins. The cleavage of fibrinogen was also investigated by incubating samples of fibrinogen with the venom and submitting them to SDS-PAGE analysis in reducing conditions. α and β chains of fibrinogen were completely cleaved in the presence of 0.5 µg of venom (Figure 2C). EDTA and PMSF completely prevented β chain degradation and showed partial inhibition of α chain cleavage, whereas 1,10-phenanthroline abolished both α and β chain cleavage.

Finally, the proteolytic activity of *B. lanceolatus* venom upon FRET peptidic substrates was investigated. In linear kinetics conditions (excess of substrate), the specific activities of *B. lanceolatus* venom on the peptides Abz-FRSSRQ and Abz-RPPGFSPFRQ were of the values 111.0 ± 4.7 and 78.3 ± 9.6 UF/min/µg, respectively (Table 2). Upon pre-incubation with inhibitors, those activities could be partially or completely abolished. Cleavage of the peptide substrate Abz-FRSSRQ could be fully inhibited by the use of PMSF, whereas full inhibition of Abz-RPPGFSPFRQ cleavage could be achieved with EDTA and 1,10-phenanthroline.

We tested the ability of Brazilian bothropic antivenom to neutralize the hyaluronidase, PLA2, and proteolytic activities of the venom. The IC95 for *B. jararaca* venom was first determined for hyaluronidase and PLA2 activities and then tested on *B. lanceolatus* venom. This concentration was sufficient to completely inhibit *B. lanceolatus* hyaluronidase activity. On the contrary, IC95 for *B. lanceolatus* PLA2 activity was found to be about seven times higher than IC95 for *B. jararaca* venom (Table 1). No complete inhibition of *B. lanceolatus* venom proteolytic activity could be reached in the fluorimetric assay (Table 2).

### 2.3. Cytotoxicity of the Venom for Human Cells

**Figure 3:**
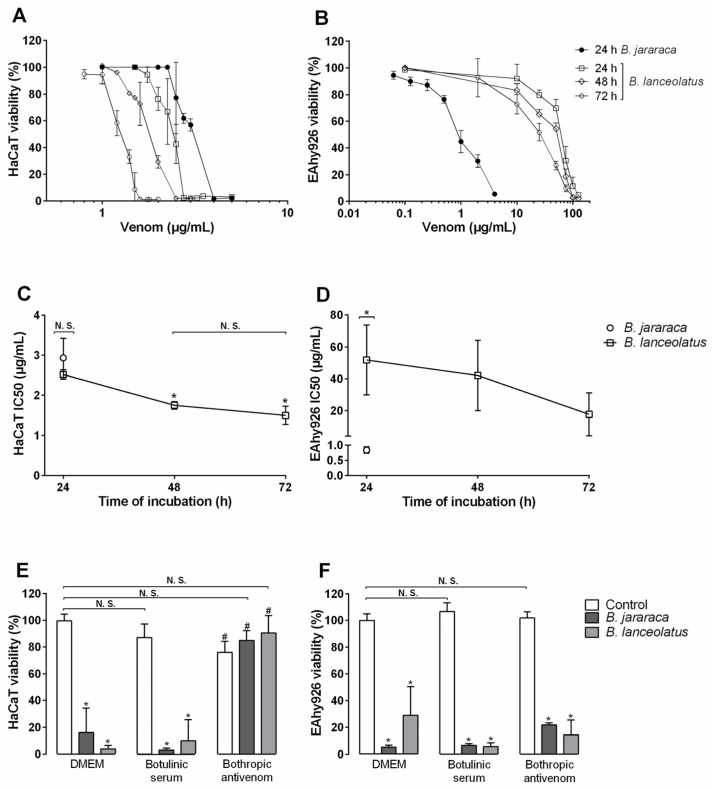
Cytotoxicity of *B. lanceolatus* venom on human keratinocytes HaCaT and endothelial vascular cells EAhy926 and its inhibition by bothropic antivenom. (**A**–**D**) After being subcultured in 96-well plates in DMEM medium, HaCaT and EAhy926 cells were exposed to different concentrations of *Bothrops* venoms or only with medium as control. Cellular viability was assessed after 24, 48, and 72 h of incubation by MTT assay. The percentage of viability and IC50 were calculated using software GraphPad Prism. The experiments were done three times, in triplicate. (**A**,**B**) are representatives of one experiment. (**C**,**D**) represent the IC50 values, calculated as the means of the three assays for each time interval, using venom concentrations represented in (**A**,**B**). (**E**,**F**) Venom samples were pre-incubated with bothropic antivenom for 30 min at room temperature. Cells were exposed to these samples for 24 h before cell viability was assessed by MTT assay. Data are represented as average ± standard error. The columns represent the mean ± SD (*n* = 3 each). NS: not significant. * *p* < 0.05 compared to the corresponding control. ^#^ *p* < 0.05 compared to the corresponding column for DMEM-treated cells without antiserum.

Incubation of human keratinocytes HaCaT with *B. lanceolatus* venom induced a loss of more than 95% of cell viability for concentrations above 2.7 µg/mL during 24 h (Figure 3A). 50% of viability was reached with the concentration of 2.5 µg/mL (CI 95: 2.3–2.7 µg/mL; Figure 3C). After 48 and 72 h of incubation, a 50% loss of viability was reached for approximately 1.7 µg/mL (CI 95: 1.6–1.9 µg/mL) and 1.5 µg/mL (CI 95: 0.9–2.0 µg/mL), respectively. After 24 h of incubation, *B. jararaca* venom presented an IC50 of 2.9 µg/mL (CI 95: 1.7–4.1 µg/mL; Figure 3A,C), which was not significantly different from *B. lanceolatus* venom IC50. The two *Bothrops* venoms presented a significative difference of cytotoxic potential for endothelial vascular cells EAhy926 (Figure 3B,D). After 24 h of exposure, concentrations of *B. jararaca* venom above 4 µg/mL induced at least 90% of viability loss, whereas the same effect was observed with a minimum of 125 µg/mL of *B. lanceolatus* venom (Figure 3B). The calculated IC50 were 0.8 µg/mL (CI 95: 0.5–1.0 µg/mL) and 51.7 µg/mL (CI 95: 0–107.2 µg/mL), for *B. jararaca* and *B. lanceolatus* venom, respectively (Figure 3D). After 48 h of exposure to *B. lanceolatus* venom; the IC50 was 42.4 µg/mL (CI 95: 0–97.5 µg/mL), and, after 72 h, it was 17.6 µg/mL (CI 95: 0–51.2 µg/mL).

Neutralization of the cytotoxicity of *B. lanceolatus* venom for the two cell lineages by bothropic antivenom was tested by preincubating venom with antivenom. *B. jararaca* venom was used as the positive control, and botulinum toxin antiserum was used as the negative control. The concentrations of venom were set so as to obtain about 100% loss of viability within 24 h. Figure 3E shows the complete inhibition of both *Bothrops* venoms’ cytotoxicity in keratinocytes by the bothropic antivenom, using 1:20 antivenom dilution. However, no inhibition of the venoms’ cytotoxicity was observed for endothelial vascular cells, using antivenom dilution up to 1:5 (Figure 3F). The negative control showed no interference of non-specific antibodies with the assay.

### 2.4. Inflammatory Potential of the Venom for Human Keratinocytes and Vascular Endothelial Cells

**Figure 4:**
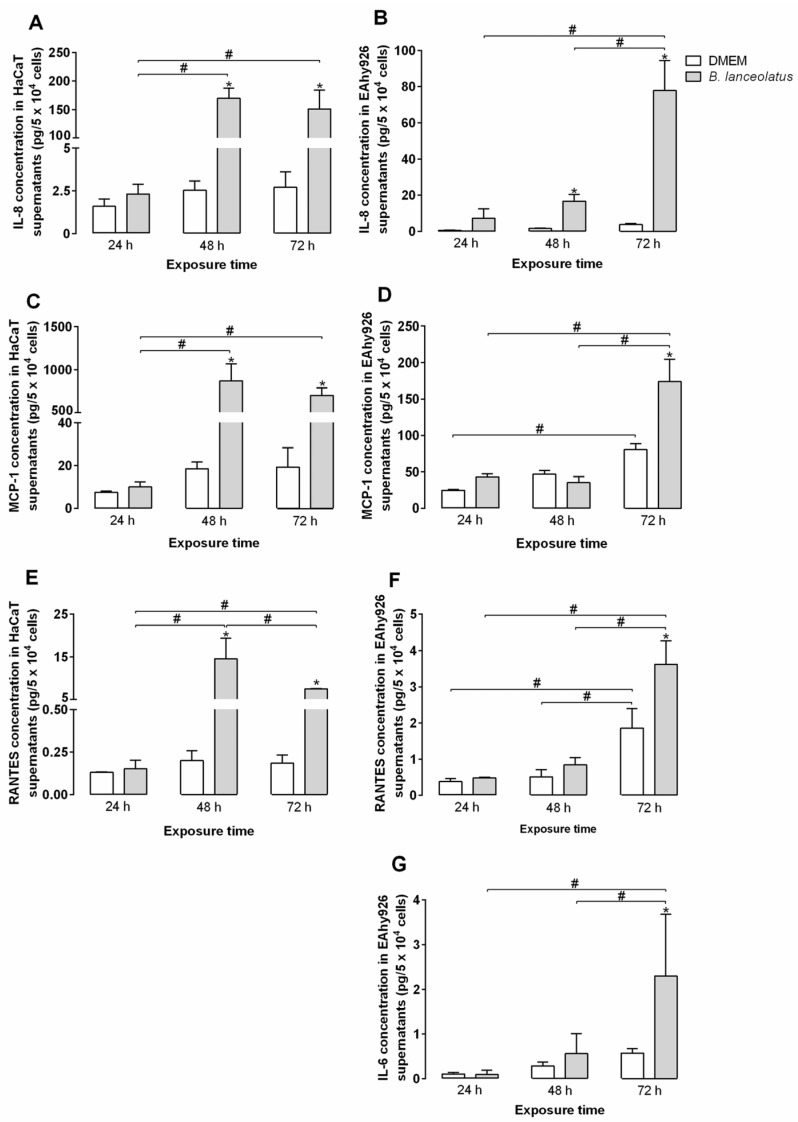
Cytokines and chemokines produced by HaCaT and EAhy926 cells incubated with *B. lanceolatus* venom for 24, 48, and 72 h. EAhy926 were treated with 50 µg/mL of *B. lanceolatus* venom (**B**,**D**,**F**,**G**), whereas HaCaT cells were incubated with 2 µg/mL of venom (**A**,**C**,**E**). After 24, 48, or 72 h, cell viability was assessed by MTT assay and supernatants were collected for cytokines and chemokines detection using Cytometric Bead Array (BD, NJ, USA). The results shown are representative of two experiments, each done in triplicate. The columns represent the mean ± SD. * *p* < 0.05 compared to the corresponding saline-treated control. ^#^ *p* < 0.05 between samples.

The effect of venom on human keratinocytes and endothelial vascular cells was further investigated by measuring the production of cytokines and chemokines by venom-treated cells in conditions of partial loss of viability. In the supernatant of venom-exposed keratinocytes and endothelial vascular cells, four cytokines and chemokines were detected: IL-8, MCP-1, RANTES, and IL-6, the latter observed only in EAhy926 supernatants (Figure 4). IP-10, MIG, IL-12p70, TNF-α, IL-10, IL-1β, IL-17a, IFN-γ, IL-4, and IL-2 were not detected in any of the culture supernatants (data not shown). Concentrations of IL-8, MCP-1, and RANTES were stable along the time in the supernatants of DMEM-treated keratinocytes (Figure 4A,C,E). From 48 h of incubation, concentrations of IL-8, MCP-1, and RANTES increased significantly in the supernatants of venom-treated keratinocytes when compared to DMEM-treated cells. Concentrations of the three proteins increased between 24 to 48 h of incubation with the venom. IL-8 and MCP-1 concentrations stayed stable between 48 and 72 h of incubation, whereas RANTES concentrations decreased in this period of time (Figure 4A,C,E).

In the endothelial vascular cells supernatants, levels of MCP-1 and RANTES increased in the control wells after 72 h of incubation, whereas IL-6 and IL-8 concentrations did not significantly change over the experiment (Figure 4B,D,F,G). A raise of IL-8 concentration was observed in the supernatants of cells treated with *B. lanceolatus* venoms for 48 h (Figure 4B). After 72 h incubation, concentrations of the four proteins were significantly higher in venom-treated cell supernatants than in the control wells (Figure 4B,D,F,G).

To ensure that venom proteolytic activity did not interfere with the assay, direct cleavage of the cytokines and chemokines by the venom was tested. It highlighted that *B. lanceolatus* venom directly cleaved MIG and IL-12p70 (Table 3) but did not display any activity upon IP-10, MCP-1, RANTES, IL-8, TNF-α, IL-10, IL-6, and IL-1β (data not shown). As shown by the use of specific protease inhibitors, MIG was mainly cleaved by SVMPs, whereas IL-12 was hydrolyzed by both SVMPs and SVSPs (Table 3).

**Figure 5:**
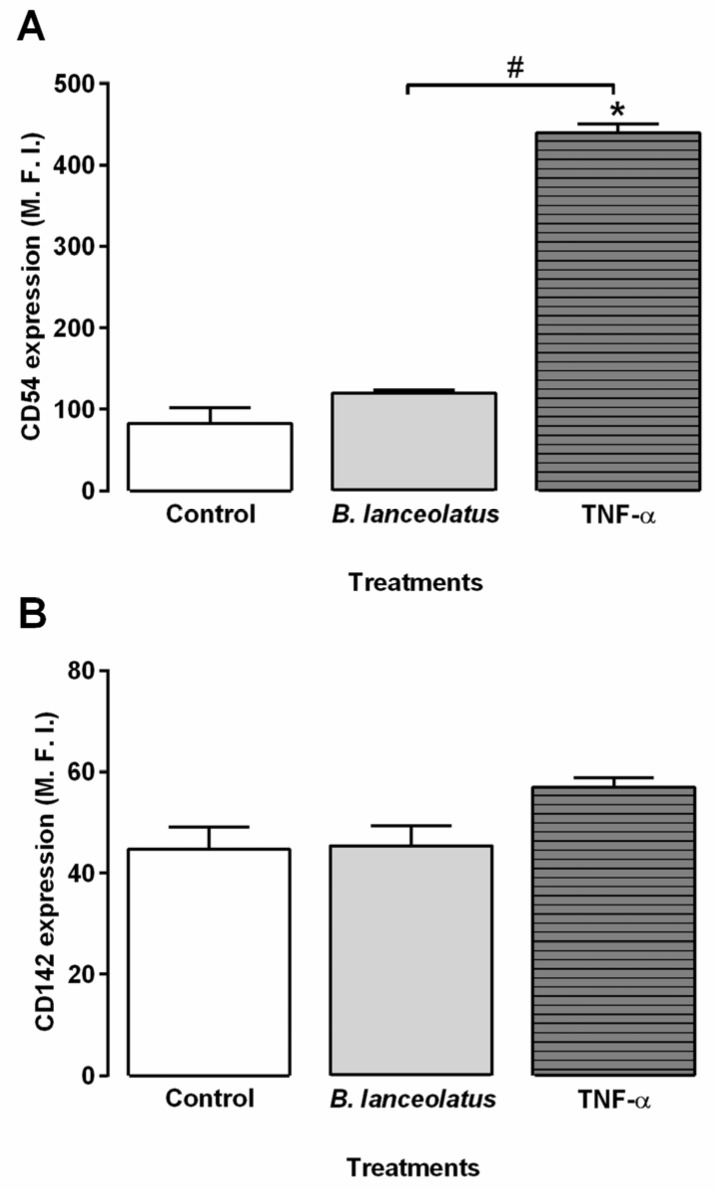
Expression of the adhesion protein, ICAM-1 (CD54), and tissue factor (CD142) on the surface of endothelial cells incubated with *B. lanceolatus* venom. EAhy926 (10^6^ cells/mL) were incubated with *B. lanceolatus* venom (100 µg/mL) or TNF-α (20 ng/mL) for 2 h. After labelling and fixing, the fluorescence of CD54 (**A**) and CD142 (**B**) was assessed by flow cytometry and expressed as the median fluorescence intensity (M. F. I.). The columns represent the mean ± SD of triplicate determinations from one experiment. This experiment was done on two separate occasions. * *p* < 0.05 compared to the saline-treated controls. ^#^ *p* < 0.05 between two samples.

The activation of vascular endothelial cells by *B. lanceolatus* venom was further explored by analyzing the modulation of adhesion molecules and tissue factor expression on vascular endothelial cell membranes. Cells were exposed for 2 h to the venom and to saline or TNF-α as the negative and positive controls for cell activation, respectively. TNF-α, but not *B. lanceolatus* venom, induced significant intensification of ICAM-1 expression on endothelial cell membranes, as shown in Figure 5. Nonetheless, tissue factor expression was not increased upon TNF-α or venom exposure. E-selectin and VCAM-1 were not detected in this immortalized cell lineage (data not shown), as already reported by Lidington et al. [25].

## 3. Discussion

In the present study, we showed that *B. lanceolatus* venom has strong phospholipase A2, gelatinolytic, and fibrinogenolytic activities. The gelatinolytic and fibrinogenolytic activities were associated with distinct enzymes, as showed by zymography assays. In the venom, both SVMPs and SVSPs cleave fibrinogen. The bidimensional electrophoretic profile of *B. lanceolatus* venom is comparable to other *Bothrops* venoms [25]. It presented a high molecular mass protein (~70 kDa) with a pI of 5, the characteristics of which correspond to class III SVMPs. Several proteins of about 30 kDa were resolved with an pI between 6.5 and 7 and could be related to SVSPs already described. Below 20 kDa appeared several acid and basic proteins, which were identified as C-type lectin-like proteins, and phospholipases in other *Bothrops* venoms [26]. Glycosylation is a post-translational modification that enhances snake venom complexity, as demonstrated with *B. jararaca* venom [27,28]. The western blot using lectins showed that several components of *B. lanceolatus* venom share this characteristic.

Hyaluronic acid is a polysaccharide of high molecular mass, which is an essential component of extracellular matrices in soft interstitial tissues. Hyaluronidase-mediated degradation of hyaluronic acid increases interstitial tissue permeability and lowers the viscosity of extracellular fluids [29], enhancing the diffusion of toxins and promoting their direct contact with cellular membranes. *B. lanceolatus* venom presented low hyaluronic activity, compared with other *Bothrops* venoms [30]. This may contribute to the low hemorrhage and necrosis observed in cases of envenomation by *B. lanceolatus*, compared to those observed in envenomations by other *Bothrops* species.

*Bothrops* venoms affect the diffusion of inflammatory mediators as they hydrolyze extracellular matrix components, but they can also display a direct hydrolytic activity upon them. A class-III SVMP, purified from *B. jararaca* venom, was shown to cleave IL-6, TNF-α, and IL-1β [31]. Here, we observed the proteolytic hydrolysis of MIG and IL-12 by *B. lanceolatus* venom. MIG was mainly cleaved by SVMPs, whereas both SVMPs and SVSPs had activity upon IL-12. MIG is a chemotactic factor for T-lymphocytes [32]. IL-12 induces the differentiation of T_H_1 lymphocytes, the maturation of cytotoxic lymphocytes, and IFN-γ production by these cells. The degradation of these two proteins could compromise the adaptive immune response involved in the production of specific antibodies but may probably not influence the envenoming acute clinical picture.

The bothropic antivenom produced against Brazilian snakes cross-reacted with *B. lanceolatus* venom and its hyaluronidase, phospholipase, and cytotoxic (on keratinocytes) activities could be fully abrogated by the antivenom. On the contrary, the inhibition of *B. lanceolatus* venom proteolytic activity and cytotoxicity for endothelial cells by the antivenom was partial, showing that some, but not all, components from *B. lanceolatus* venom share antigenic similarities with other *Bothrops* species from South America. Abz-FRSSRQ substrate is cleaved mainly by serine proteases in *B. lanceolatus* venom, whereas both serine proteases and metalloproteinases act on Abz-RPPGFSPFRQ. However, in the study by Kuniyoshi et al. [33], Abz-RPPGFSPFRQ substrate was mainly cleaved by SVSPs from South American *Bothrops* venoms. These data suggest that *B. lanceolatus* proteases present different substrate specificity from their continental bothropic counterparts.

Several studies addressing the cytotoxic action of venoms of the *Bothrops* species from South America or the cytotoxicity of proteins purified from these venoms for endothelial cells have been performed [34,35,36,37,38,39,40,41,42,43,44,45], but there are few reports of *Bothrops* venom toxicity for keratinocytes [46,47]. To date, nothing is known about the cytotoxic potential of *B. lanceolatus* venom. Two human immortalized cell lines were chosen to investigate venom-induced local/systemic inflammation: HaCaT keratinocytes and EAhy926 vascular endothelial cells. In the assay conditions, the venom affected keratinocyte viability from 2 µg/mL concentration and 24 h of exposure. With similar exposure time, *B. jararaca* venom was as toxic as *B. lanceolatus* venom, demonstrating a high toxicity of both *Bothrops* venoms in this cell line. However, both venoms displayed different cytotoxic patterns on EAhy926 endothelial cells. After 24 h incubation, *B. lanceolatus* venom appeared about 10 times less toxic than *B. jararaca* venom. Longer times of incubation enhanced *B. lanceolatus* venom cytotoxicity. Interestingly *B. lanceolatus* venom results reproduced the in vivo observations of Jiménez et al [45]; in a murine model of cutaneous tissue damage, class I SVMP, BaP1, causes the apoptosis of keratinocytes without affecting the viability of endothelial cells. As EAhy926 cells present more chromosomes than human umbilical vein endothelial cell (HUVECs), which could influence cells’ resistance to venom exposure, these results could be confirmed using HUVECs [25,48].

Keratinocytes play an important part in the inflammatory process by producing a wide variety of cytokines. Once activated, they also produce chemokines, which attract leucocytes to the inflammation spot [49]. In venom-exposed HaCaT supernatants, three chemokines were detected: IL-8, MCP-1, and RANTES. The main function of IL-8 (or CXCL-8) is the recruitment of leukocytes, especially neutrophils, whereas MCP-1 (CCL-2) and RANTES (CCL-5) attract monocytes and leukocytes, respectively. The concentration of the three cytokines, IL-8, MCP-1, and RANTES, increased in venom-treated cell supernatants after 48 h incubation with the venom, indicating direct inflammatory stimulus for the cells.

Endothelial vascular cells also play a key role in inflammation. They control inflammatory cell adhesion and migration from the bloodstream to damaged tissues, as well as fluid exchange between the two compartments. In supernatants of EAhy926 endothelial cells exposed to *B. lanceolatus* venom, we detected four cytokines: IL-6, MCP-1, RANTES, and IL-8. The concentrations of these proteins increased in venom-exposed cell supernatants after 48 or 72 h of venom exposure. In order to complete our study, we analyzed activation markers expression such as adhesion protein ICAM-1 and tissue factors on venom-exposed endothelial cell membranes, using flow cytometry. ICAM-1 expression was significantly augmented by TNF-α exposure, whereas *B. lanceolatus* venom did not significantly alter its expression. In vivo, *B. asper* venom stimulates endothelial cells to express adhesion molecules such as ICAM-1 [50], whereas *B. jararaca* venom influence the expression of selectin and integrin genes in HUVECs [43,51]. The present authors suggest that toxin destabilizes cells, which could turn it more sensitive to inflammatory mediators from cellular microenvironments. This hypothesis could explain the absence of modulations in ICAM-1 expression observed in our model and of the expression of certain inflammatory cytokines.

## 4. Conclusions

The data presented here expose new features of *B. lanceolatus* venom such as low hyaluronidase activity, protease substrate specificity, and cytotoxicity for keratinocytes and endothelial cells and bring insights into antigenic similarity between *B. lanceolatus* venom toxins and South American *Bothrops* venoms toxins. Our in vitro model of venom-induced cytokine and chemokine production reveals the high pro-inflammatory potential of the venom in human cell lines. Further describing *B. lanceolatus* venom interactions with cytokines and human cell lines that are relevant in inflammation processes could help to improve envenomation management.

## 5. Materials and Methods

### 5.1. Chemicals and Reagents

Triton X-100, Tween 20, Brij-35, EDTA, *N*-Cetyl-*N*,*N*,*N*-trimethylammonium bromide (CTAB), ortho-phenylenediamine (OPD), Coomassie Blue R-250, hyaluronic acid, diaminobenzidine (DAB), phenylmethanesulfonyl fluoride (PMSF), 1,10-phenanthroline, bovine serum albumin (BSA), fibrinogen from human plasma, gelatin type A, goat anti-horse (GAH) IgG horseradish peroxidase (IgGHRPO), concanavalin A (Con A), and Wheat Germ Agglutinin (WGA) labelled with peroxidase, were purchased from Sigma-Aldrich (St. Louis, MO, USA). Dithiothreitol (DTT) and iodoacetamide were obtained from GE Healthcare Life Sciences (Pittsburgh, PA, USA). 3-(4,5-dimethylthiazol-2-yl)-2,5-di-phenyltetrazolium bromide (MTT) and dimethyl sulfoxide (DMSO) were from Merck (Darmstadt, Germany). GAH IgG labelled with alkaline phosphatase (IgG-AP), 5-bromo-4-chloro-3-indolyl-phosphate (BCIP), and nitroblue tetrazolium (NBT) were from Promega Corp. (Madison, WI, USA). Fluorescent resonance energy transfer (FRET) substrates Abz-FRSSRQ-EDDnp (Abz-FRSSRQ) and Abz-RPPGFSPFRQ-EDDnp (Abz-RPPGFSPFRQ) were obtained from GenOne Biotechnologies (Rio de Janeiro, RJ, Brazil).

### 5.2. Venoms

Venom from *Bothrops lanceolatus* was obtained from Latoxan (Aix-en-Provence, France). Venoms from *Bothrops jararaca* and *Crotalus durissus terrificus* were supplied by the Herpetology Laboratory of the Butantan Institute. Stock solutions were prepared in sterile saline solution (150 mM NaCl) at 5 mg/mL and stored at −80 °C.

### 5.3. Sera

The bothropic antivenom and botulinum toxin antiserum, both containing only purified immunoglobulin F(ab′)2 fraction, were kindly donated by the Hyperimmune Plasma Processing Section of the Butantan Institute (São Paulo, Brazil).

### 5.4. Unidimensional Electrophoresis and Western Blotting

*B. lanceolatus* venom was solubilized in non-reducing or reducing sample buffer, incubated at 96 °C for 5 min. Then samples were separated by SDS-PAGE on 12% acrylamide gels [52], stained with Coomassie blue or blotted onto nitrocellulose [53]. After transfer, the membranes were blocked with phosphate buffer saline (PBS; 8.1 mM Na_2_HPO_4_, 1.5 mM KH_2_PO_4_, 137 mM NaCl and 2.7 mM KCl, pH 7.2) containing 5% BSA and incubated with bothropic antivenom (1:10,000) for 1 h at room temperature. Immunoreactive proteins were detected using GAH/IgG-AP (1:7500) in PBS/1% BSA for 1 h at room temperature. After washing three times for 10 min with PBS/0.05% Tween 20, blots were developed using NBT/BCIP according to the manufacturer’s instructions (Molecular Probes, Carlsbad, CA, USA). For the western blot using lectins, after transfer and blocking, the membranes were incubated with Con A or WGA labelled with peroxidase for 1 h at room temperature. The membranes were then washed with PBS containing Tween-20 (0.05%) and developed with DAB (20 mg/mL).

### 5.5. Bi-Dimensional Electrophoresis

Precast IPG strips (13 cm, pH 3–10, linear, GE Healthcare) were rehydrated with 250 µL of DeStreak rehydration solution (GE Healthcare) containing 1% IPG buffer (GE Healthcare) and 100 µg of *B. lanceolatus* venom for 16 h at room temperature. First dimension separation was carried out using an Ettan IPGphor Isoelectric Focusing System (GE Healthcare), following th emanufacturer’s instructions. We followed a five-phase electrophoresis program; a step of 500 V for 60 min, a gradient of 1000 V for 60 min, a gradient of 8000 V for 2 h 30 min, a step of 1000 V for 12 h (so as to run overnight), and finally a step of 8000 V for 30 min. Right at the end of the separation, the IPG strips were sequentially incubated in equilibration buffer (50 mM Tris-HCl, pH 8.8, 2% SDS, 6 M urea, 30% glycerol, bromophenol blue) containing 10 mg/mL DTT for 15 min and then in equilibration buffer containing 25 mg/mL iodoacetamide for 15 min. After that, they were briefly washed in electrophoresis buffer (25 mM Tris, 192 mM glycine, 0.1% SDS) and applied to 12% SDS-polyacrylamide gels (10 × 10 cm) for second dimension electrophoresis, using the Hoefer Mini VE apparatus (GE Healthcare). The gels were finally silver-stained.

### 5.6. Enzyme Linked Immunosorbent Assay (ELISA)

Microtitre plates were coated with *B. lanceolatus* venom (10 µg/mL in 100 µL PBS; overnight at 4 °C) and an ELISA assay was performed with bothropic antivenom, as previously described [29]. Botulinum toxin antiserum was used as the negative control. The absorbance was recorded at 492 nm using a microplate reader (Multiskan spectrophotometer EX, Vantaa, Finland). The highest antivenom dilution producing an absorbance eight times greater than that determined for the negative control serum was defined as the antiserum titer.

### 5.7. Phospholipase A2 Activity

The PLA2 activity of *B. lanceolatus* venom was investigated using an EnzChek^®^ Phospholipase A2 Assay Kit (Invitrogen, Paisley, UK), following the manufacturer’s instructions. Venoms from *B. jararaca* and *C. durissus terrificus* were used as the positive controls. The venom concentrations were chosen according to preliminary dose-response curves. Fluorescence measurements were performed at 37 °C using the spectrometer FLUOstar Omega (BMG Labtech, Offenburg, Germany) at the wavelength λ_EM_ = 515 nm, with an excitation at λ_EX_ = 485 nm every 30 s for 5 min. Specific activity was expressed as the units of free fluorescence of cleaved substrate/min/µg of venom.

### 5.8. Hyaluronidase Activity

The hyaluronidase activity of the venom was measured following the procedures of Pukrittayakamee et al. [54]. The venom concentration was chosen according to the preliminary dose-response curve. Briefly, after incubation with hyaluronic acid, the samples were mixed with CTAB-containing solution (2.5% CTAB and 2% NaOH). CTAB forms a complex with the remaining hyaluronic acid, thereby allowing absorbance measurement. The turbidity measurements were determined in an ELISA plate reader (Multiskan EX, Labsystems, Vantaa, Finland) at 405 nm. Hyaluronidase activity was expressed in TRU (turbidity reducing units)/mg; one TRU corresponds to the amount of venom required to hydrolyze 50% of hyaluronic acid.

### 5.9. Cleavage of Fibrinogen

The venom samples were pre-incubated or not with 20 mM of inhibitors (PMSF, 1,10-phenanthroline or EDTA). To evaluate *B. lanceolatus* venom’s fibrinogenolytic activity, samples of fibrinogen (30 µg) were incubated with the venom for 30 min at 37 °C and submitted to electrophoretic separation in reducing conditions.

### 5.10. Zymography

*B. lanceolatus* venom’s gelatinolytic and fibrinogenolytic activities were analyzed by zymography. Venom samples (25 µg) were solubilized in normal sample buffer and separated on gels containing 10% of polyacrylamide and 1 mg/mL of gelatin type A or fibrinogen from human plasma. The separation was realized under constant tension (80 V) and temperature (4 °C) in the Mighty Small system (Hoefer Pharmacia Biotech, San Francisco, CA, USA). After separation, the gelatin gels were washed once for 30 min in Triton X-100 (2.5%) at room temperature, whereas the fibrinogen-containing gels were submitted to this wash three times. The gels were incubated overnight at 37 °C in substrate buffer (50 mM Tris-HCl, 200 mM NaCl, 10 mM CaCl_2_ and 0.05% Brij-35; pH 8.3). They were finally stained in Coomassie Brilliant Blue R-250 (0.2%) for 30 min under constant agitation in order to reveal the bands where proteolytic digestion occurred.

### 5.11. Fluorescent Peptidic Substrates

The fluorescence resonance energy transfer (FRET) substrates, Abz-FRSSRQ and Abz-RPPGFSPFRQ, were used to study *B. lanceolatus* venom’s proteolytic activity in the presence or absence of inhibitors and bothropic antivenom, following the procedures described by Queiroz et al. [29]. Abz-RPPGFSPFRQ was chosen because it is specifically cleaved by *Bothrops* venom SVSPs [32]. Based on preliminary experiments, venom concentrations were chosen to obtain linear kinetics, i.e., 50 µg/mL of venom for the Abz-RPPGFSPFRQ substrate and 25 µg/mL of venom for the Abz-FRSSRQ substrate. Samples containing PMSF, 1,10-phenanthroline, both diluted in ethanol, and their corresponding control samples (same volume of ethanol) were preincubated 30 min at room temperature with the venom before the addition of substrates. EDTA (100 mM) was mixed with the venom prior to the addition of the substrate without preincubation. The assays were conducted in 100 µL of phosphate buffer (50 mM phosphate, 20 mM NaCl, pH 7.4), containing the peptide substrates in a final concentration of 5 µM on Corning^®^ 96-well plates. The reactions were monitored at 37 °C (λ_EM_ = 420 nm and λ_EX_ = 320 nm) in a fluorescence spectrophotometer (FLUOstar Omega, BMG Labtech, Offenburg, Germany). The specific activity was expressed as units of free fluorescence (UF) of cleaved substrate/min/µg of venom.

### 5.12. Neutralization of B. lanceolatus Venom Enzymatic Activities

The ability of bothropic antivenom to neutralize the PLA2, hyaluronidase, and proteolytic activities of *B. lanceolatus* venom was investigated using pre-incubation protocols. Venom samples and antivenom were incubated at room temperature for 30 min, and the residual enzymatic activity was measured as described above. Initially, the amount of antivenom that inhibited 95% of the enzymatic activity of interest in *B. jararaca* venom (IC95) was determined. This amount of antivenom was then tested against *B. lanceolatus* venom. If complete inhibition was not achieved, then we determined the IC95 for the inhibition of this activity. It was not possible to determine the IC95 in the proteolytic assay because of interference by the antivenom. In this case, venom samples (5 µg or 2.5 µg depending on the substrate) were incubated with the maximum volume of bothropic antivenom (20 µL) that would not interfere with the measurements.

### 5.13. Cytoxicity in Human Keratinocytes and Vascular Endothelial Cells

The human keratinocyte HaCaT cell line was obtained from the ‘Banco de Células do Rio de Janeiro’ (Rio de Janeiro, Brazil). The cells were kept in Dulbecco’s modified eagle medium (DMEM; Gibco, Invitrogen Corp., Carlsbad, CA, USA) containing 10% (*v*/*v*) heat-inactivated fetal bovine serum (FBS; Cultilab, São Paulo, Brazil) and the following antibiotics; penicillin and streptomycin (100 IU/mL) (Gibco, Invitrogen Corp., CA, USA). Cells were grown at 37 °C in humidified air with 5% CO_2_. To assess the cell viability upon venom exposure, the colorimetric MTT assay adapted from Mosmann [55] was used. After having been sub-cultured in 96-well plates (5 × 10^4^ cells/well for HaCaT cells; 10^4^ cells/well for EAhy926; 200 µL/well) and maintained in FBS-containing medium for 24 h and in medium without FBS for an additional 24 h, the cells were then exposed to the venom for 24, 48, and 72 h. DMEM without FBS was used as the control. At the end of the exposure, the supernatant was collected. Endothelial cells were incubated with MTT (0.5 mg/mL in 200 µL DMEM) for 4 h at 37 °C (5% CO_2_), whereas keratinocytes, which metabolize MTT more rapidly, were incubated with 0.83 mg/mL of MTT solution (60 µL/well) for 30 min. After removing the MTT solution, 100 µL of DMSO was added to each well. The absorbance was measured in a spectrophotometer (Multiskan-EX, Labsystems, Finland) at 540 and 620 nm. The relative cell viability was calculated as follows:

$$\mathrm{cell}\;\mathrm{viability}\;\left(\%\right)=\;100\times \frac{\left[{\mathrm{Abs}}_{540}-{\mathrm{Abs}}_{620}\right]\left(\mathrm{sample}\right)}{\left[{\mathrm{Abs}}_{540}-{\mathrm{Abs}}_{620}\right]\left(\mathrm{control}\right)}$$

After plotting cell viability as a function of the venom concentration logarithm, non-linear regression was performed using Prism6 software (GraphPad Software, La Jolla, CA, USA) to calculate the concentration able to impair cell viability by 50% (IC50).

### 5.14. Neutralization of Venom Cytotoxicity by Bothropic Antivenom

The inhibition of *B. lanceolatus* venom cytotoxicity for human cells was assessed by MTT assay, as described above. The venom samples were previously incubated with the antivenom for 30 min at room temperature and incubated with the cells for 24 h. *B. jararaca* venom was used as the positive control, and botulinum toxin antiserum was used as the negative control. Keratinocytes were incubated with a venom concentration of 4 µg/mL. Due to the difference in cytotoxic potential for endothelial cells between the two venoms, these cells were incubated with final concentrations of 125 µg/mL of *B. lanceolatus* venom and 10 µg/mL of *B. jararaca* venom.

### 5.15. Cytokine and Chemokine Release by Venom-Treated Cells

The cell culture supernatants collected from the cell viability assay were centrifuged at 400× *g* and stored at −80 °C until use. The presence of cytokines and chemokines in the samples was assessed using ‘Cytometric Bead Arrays’ (CBA) with the ‘Human Chemokine Kit’, ‘Human Inflammatory Cytokine Kit’, and ‘Human Th1/Th2/Th17 Kit’ (BD Biosciences, Franklin Lakes, NJ, USA), following the manufacturer’s instructions.

In order to evaluate a potential direct cleavage of these cytokines and chemokines by *B. lanceolatus* venom, which could interfere with the analysis of cells supernatants, samples of cytokines and chemokines standard mixtures were incubated with the venom for 30 min at 37 °C in the presence or absence of proteases inhibitors (PMSF, EDTA, and 1,10-phenanthroline; 20 mM). The samples were subsequently submitted to ‘Cytometric Bead Arrays’, following the manufacturer’s instructions. Concentrations of cytokines and chemokines were calculated in pg/mL of supernatant from the standard linear regression curves, using FCAP Array 3.0 software (Becton Dickinson, San Jose, CA, USA).

### 5.16. Expression of Membrane Proteins by Vascular Endothelial Cells Exposed to the Venom

Endothelial cells (10^6^ cells/mL) were incubated with 100 µg/mL of *B. lanceolatus* venom for 2 h at 37 °C under constant agitation. TNF-α (20 ng/mL; BD Pharmingen, Franklin Lakes, NJ, USA) and saline solution were used as the positive and negative controls, respectively. The cells were centrifuged at 400× *g* and resuspended in FACS buffer (PBS containing 1% BSA and 0.01% NaN_3_). Then, cells (5 × 10^4^ cells/well; total volume of 25 µL) were incubated for 1 h at 4 °C, with PE-conjugated murine anti-tissue factor (TF, CD142) antibodies (BD Pharmingen, NJ, USA; diluted 1:2.5). Alternatively, the cells were incubated (5 × 10^4^ cells/well; total volume of 50 µL) for 1 h at 4 °C with the following antibodies: murine monoclonal antibodies anti-intercellular adhesion molecule 1 (ICAM-1 or CD54; Santa Cruz Biotechnology, Dallas, TX, USA; diluted 1:400), monoclonal murine antibodies donated by Dr Vaclav Horeji (Prague, Czech Republic) against vascular cell adhesion molecule 1 (VCAM-1), and E-selectin (CD62E), diluted 1:50. After centrifugation (320× *g*; 5 min) and removal of the supernatant, cells were resuspended in FACS buffer and incubated with secondary antibodies, diluted in the ratio 1:100 for 1 h at 4 °C, and protected from light (Goat Anti-Mouse IgG-PE, Sigma, St. Louis, MO, USA). The samples incubated with isotype-matched, non-specific antibodies (BD Pharmingen, San Jose, CA, USA), or only with secondary antibodies were assayed in parallel as controls. All antibodies used were previously titrated. After washing, cells were resuspended in 200 µL of FACS buffer and analyzed by flow cytometry (FACSCanto-II, BD Biosciences, NJ, USA) using FACSDiva software (BD Biosciences, NJ, USA). After gate definition, 5000 events per sample were analyzed and the fluorescence median was calculated.

### 5.17. Statistics

Numerical data were expressed as mean ± SD. Statistical comparisons were done with one-way ANOVA and Bonferroni’s multiple comparisons test, using Prism v6 (GraphPad Software, Inc., USA). A *p*-value of <0.05 was considered significant.

## Figures and Tables

**Table 1:** Hyaluronidase and phospholipase activities of *B. lanceolatus* venom; specific activities and neutralization by bothropic antivenom.

Enzymatic Activities	*C. durissus terrificus*	*B. jararaca*	*B. lanceolatus*
**Hyaluronidase**			
Specific activity (TRU/mg)	NT	116.6 ± 8.6	20.5 ± 2.4 *
Amount of bothropic antivenom (mL/mg of venom) required to inhibit activity by 95%	NT	0.056	≤0.056
**Phospholipase**			
Specific activity (UF/min/µg)	461.4 ± 11.7	121.9 ± 6.9 ^#^	231.3 ± 6.4 *
Amount of bothropic antivenom (mL/mg of venom) required to inhibit activity by 95%	NT	0.32	2.29

NT: not tested; * *p* < 0.05 compared to *B. jararaca* venom; ^#^ *p* < 0.05 compared to *C. d. terrificus* venom.

**Table 2:** Proteolytic activity of *B. lanceolatus* venom on FRET peptidic substrates.

		Fluorescent Peptides	
		Abz-FRSSRQ	Abz-RPPGFSPFRQ
**Venom activity (UF/min/µg)**		111.0 ± 4.7	78.3 ± 9.6
**Inhibition (%)**	EDTA (100 mM)	0.8 ± 1.7	94.1 ± 6.8 **
	1,10-phenanthroline (5 mM)	4.9 ± 4.6	100 ± 0 *
	PMSF (5 mM)	93.5 ± 4.5 *	79.5 ± 13.8 *
	Bothropic antivenom	33.5 ± 33.6 *	60.8 ± 21.2 *

Venom samples (50 µg/mL for Abz-RPPGFSPFRQ substrate; 25 µg/mL for Abz-FRSSRQ substrate) were incubated with inhibitors or bothropic antivenom (200 µL/mL) for 30 min at room temperature. Then substrate (5 µM) was added to the wells and the venom activity was measured at 37 °C for 15 min by spectrophotometry. The data represent the mean ± SD (*n* = three assays, each done in duplicate for the inhibitors and *n* = six assays for the neutralization experiments). * *p* < 0.05 compared to the activity of venom alone.

**Table 3:** Cleavage of MIG and IL-12 by *B. lanceolatus* venom.

		Cytokine/Chemokine	
		IL-12p70	MIG
**Venom activity (pg/µg of venom)**		16.9 ± 9.2	3.0 ± 0.2
**Inhibition (%)**	EDTA (20 mM)	97.0 ± 5.9 *	74.3 ± 7.6 *
	1,10-phenanthroline (20 mM)	86.9 ± 16.1 *	98.1 ± 7.9 *
	PMSF (20 mM)	62.3 ± 32.2 *	0.0 ± 0.0

* *p* < 0.05 compared to the activity of venom alone.

## Abbreviations

BCIP, 5-bromo-4-chloro-3-indolyl-phosphate; BSA, Bovine Serum Albumin; CBA, Cytometric Bead Arrays; CD62E, E-selectin; CI95, Confidence interval 95%; Con A, Concanavalin A; CTAB, *N*-Cetyl-*N*,*N*,*N*-Trimethylammonium Bromide; DAB, Diaminobenzidine; DMEM, Dulbecco’s modified eagle medium; DMSO, Dimethyl Sulfoxide; DTT, Dithiothreitol; EDTA, Ethylenediamine Tetraacetic Acid; FBS, Fetal Bovine serum; FRET, Fluorescent Resonance Energy Transfer; GAH, Goat Anti-Horse; HUVECs, human umbilical vein endothelial cells, IC50, concentration able to impair cell viability of 50%; IC95, quantity of antivenom that inhibited 95% of the enzymatic activity of interest; ICAM-1, Intercellular Adhesion Molecule 1 (CD54); IFN-γ, γ-Interferon; IgG-AP, IgG labelled with Alkaline Phosphatase; IgG-HRPO, IgG Horseradish Peroxidase; IL, Interleukins; IP-10, γ-interferon-induced protein 1 (CXCL-10); MCP-1, monocyte chemoattractant protein 1 (CCL-2); MIG, monokine induced by γ-interferon (CXCL-9); *M_r_*, relative molecular mass; MTT, 3-(4,5-dimethylthiazol-2-yl)-2,5-di-phenyltetrazolium bromide; NBT, nitroblue tetrazolium; PBS, Phosphate Buffer Saline; PE, Phycoerythrin; pI, isoelectric point; PLA2, Phospholipase A2; PMSF, Phenylmethanesulfonyl Fluoride; RANTES, regulated on activation, normal T cell expressed and secreted protein (CCL-5); SVMP, Snake Venom Metalloprotease; SVSP, Snake Venom Serine Proteinase; TNF-α, Tumor Necrosis Factor-α; TRU, Turbidity Reducing Units; UF, unit of fluorescence; VCAM-1, Vascular Cell Adhesion Molecule 1; WGA, Wheat Germ Agglutinin.
